# Long-Chain n-3 Polyunsaturated Fatty Acids Attenuate the Severity of Obesity-Associated White Adipose Tissue and Skeletal Muscle Dysfunction

**DOI:** 10.3390/md24050166

**Published:** 2026-05-06

**Authors:** Ala Alzubi, Alyssa Lucchesi, Jessie L. Burns, Clara E. Cho, David W. L. Ma, Lindsay E. Robinson, Jennifer M. Monk

**Affiliations:** Department of Human Health Sciences, University of Guelph, Guelph, ON N1G 2W1, Canada; aalzubi@uoguelph.ca (A.A.); alucches@uoguelph.ca (A.L.); jessielburns-phd@outlook.com (J.L.B.); claracho@uoguelph.ca (C.E.C.); davidma@uoguelph.ca (D.W.L.M.)

**Keywords:** n-3 polyunsaturated fatty acids (PUFAs), eicosapentaenoic acid (EPA), docosahexaenoic acid (DHA), obesity, adiposity, inflammation, adipokines, adipose tissue, skeletal muscle, myokines, immune cell chemotaxis, metabolic dysfunction, insulin resistance

## Abstract

Obesity is a complex metabolic disorder defined by a body mass index greater than 30 kg/m^2^, excess adipose tissue accumulation, chronic low-grade inflammation and metabolic dysfunction, leading to increased susceptibility to other chronic conditions. Evidence from both mechanistic pre-clinical studies and human clinical trial suggests a role for long-chain (LC) n-3 polyunsaturated fatty acids (PUFAs), namely eicosapentaenoic acid (EPA) and docosahexaenoic acid (DHA), to modulate obesity-associated outcomes and attenuate the production of inflammatory mediators, namely adipokines from adipose tissue and myokines from skeletal muscle, that can mitigate inflammation-associated metabolic dysfunction. Therefore, this narrative review synthesizes evidence and mechanistic insights on the role(s) of n-3 PUFA in regulating obesity-associated changes in adipose tissue and skeletal muscle inflammation and immune cell trafficking and metabolic outcomes.

## 1. Introduction

The World Health Organization defines obesity as an excess accumulation of adipose tissue (AT) that puts the individual’s health at risk, as classified by a body mass index (BMI) that is greater than 30 kg/m^2^ [1]. In Canada, as of 2019 the prevalence of obesity in adults was 24.3%, which was characterized as moderate in comparison to other countries with both higher and lower prevalences [2]. Global pooled estimates of overweight and excess weight in children and adolescents were recently reported at 14.8% [3], wherein the prevalence varies across countries. In Canada, approximately 25% of four- to 11-year-olds and 33% of 12- to 17-year-olds have an elevated BMI that may indicate overweight or obesity [4,5]. This is relevant given that obesity status is retained into adulthood for approximately 80% of obese adolescents [6], indicative of a lifelong ongoing health challenge.

Obesity is a well-known risk factor for a number of comorbidities, including, but not limited to, cardiovascular disease, type 2 diabetes mellitus (T2D), chronic kidney disease, and certain types of cancer [7,8,9,10]. In this connection, a recent meta-analysis of observational studies has shown that every 5 kg/m^2^ increase in BMI is associated with a 10% increase in haemorrhagic stroke and a 49% increase for hypertension [11]. Chronic low-grade inflammation is a defining feature of the obese phenotype that links changes in adipose tissue (AT) function and the increased production of adipokines (mediators produced from AT) to both local and systemic metabolic dysfunction, including the development of insulin resistance and the progression to T2D [12,13,14]. Therefore, a critical intervention strategy to limit obesity severity is to target AT inflammation.

**Figure 1:**
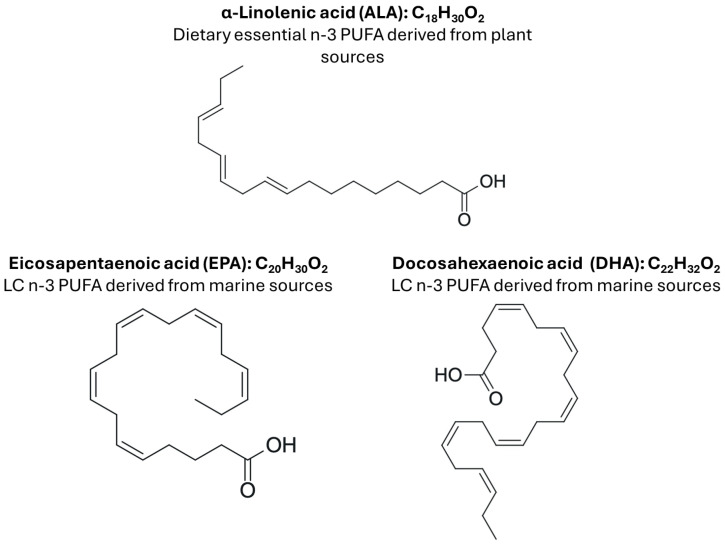
Chemical structures of plant- and marine-derived dietary sources of n-3 PUFA.

Consuming a pro-inflammatory diet may play a role in exacerbating the effects of obesity-associated chronic inflammation and is associated with elevated levels of circulating inflammatory markers in the blood, thus contributing to the increased risk of obesity-associated chronic diseases [15,16,17]. When considering the relationship between diet, obesity, and inflammation, dietary fatty acids (FAs) may play a key role. FAs play a number of critical roles in biological structure and function. Structurally, they are involved in the formation of nearly all tissues, through their incorporation into phospholipids that comprise the phospholipid bilayer of cell membranes. Functionally, they are involved in nutrient metabolism as well as numerous cell signaling pathways responsible for essential biological processes. Of these FAs, n-3 polyunsaturated fatty acids (PUFAs) (Figure 1) are necessary for normal growth and development, cellular function and signaling, and immune response, and may be critical for the prevention/attenuation of chronic inflammation and chronic disease severity, as reviewed elsewhere [18,19,20].

The three main n-3 PUFAs found in the diet include α-linolenic acid (ALA; 18:3n-3), a dietary essential fatty acid that can be sourced from the green part of plants, seeds, and nuts (e.g., flaxseed and walnuts) [21,22], and the long-chain (LC) n-3 PUFA, namely eicosapentaenoic acid (EPA; 20:5n-3) and docosahexaenoic acid (DHA; 22:6n-3), which are found in fish and seafood [23]. Despite the critical roles of n-3 PUFA in health and disease prevention, the typical North American intake of LC n-3 PUFA is low (0.1–0.2 g/day compared to the 0.5 g of EPA and DHA per day recommended by the Academy of Nutrition and Dietetics) [24,25]. Therefore, increasing LC n-3 PUFA intake (either through increased intake of dietary sources rich in LC n-3 PUFA or supplements) could function to decrease obesity severity. In this review, we discuss the role of LC n-3 PUFA in modulating obesity-associated outcomes and biomarkers of obesity-associated comorbidities, with an emphasis on the ability of LC n-3 PUFA to attenuate inflammatory mediator (adipokines in AT and myokines in skeletal muscle) production that mechanistically connects chronic inflammation to metabolic dysfunction.

## 2. Obesity-Associated Changes in AT

Adipocytes can be characterized as white, brown or beige/brite (brown-in-white, indicating white adipocytes differentiated into brown-like adipocytes within white AT), which exhibit functional and phenotypic differences [26,27]. Although all adipocyte types are involved in endocrine signaling, brown adipocytes are associated with thermogenesis, beige adipocytes are associated with adaptive thermogenesis and white adipocytes are associated with energy storage [27]. Therefore, white AT (WAT) is the primary site of lipid storage in the form of triacylglyceride following excess energy intake and is responsible for the quick mobilization of lipid stores when energy is required [26]. WAT can be divided further into two main depots: subcutaneous, which corresponds to AT located under the skin, and visceral, corresponding to AT that surrounds the internal organs [28]. Subcutaneous depots are generally considered to be a safer long-term energy storage depot compared to visceral AT, which increases in size and develops metabolic dysfunction in obesity, leading to inflammation, dyslipidemia and insulin resistance [26,29,30,31,32]. Thus, WAT plays an important role in the regulation of whole-body metabolic homeostasis in both healthy/unchallenged and diseased states, wherein it serves as a primary site for excess energy storage and performs fundamental functions [33,34,35,36]. These functions include the secretion of a variety of adipokines, which are bioactive molecules secreted by AT that can have various effects on physiological processes, including, but not limited to, the regulation of inflammation, metabolism, appetite, cardiovascular function and immunity [26,33,37].

WAT undergoes several obesity-associated changes. As a consequence of overnutrition, excess energy is stored as triglycerides in AT, which undergoes dynamic changes in the transition from a non-obese to obese state. To accommodate increased triglyceride storage, adipocytes undergo hypertrophy (increase in cell size) and hyperplasia (increase in cell number) and AT accumulates in both subcutaneous and visceral AT depots [38,39,40,41]. In humans with obesity, omental adipocyte size has been shown to positively correlate with insulin resistance [42]. It is estimated that 10–25% of individuals with obesity are metabolically healthy and retain sensitivity to insulin signaling [43]; however, these individuals are still at risk for adverse cardiovascular outcomes [44]. Furthermore, the impaired capacity for triglyceride storage in AT can result in ectopic fat deposition in other tissue sites, impairing tissue function and contributing to the development of insulin resistance [45].

The cellular composition of AT also changes in obesity, with increased immune cell recruitment to the tissue. The dominant immune cell populations in lean AT include M2-polarized macrophages (also referred to as ‘alternatively activated’), T helper (Th)2 and regulatory T cells that contribute to the maintenance of AT insulin sensitivity through the secretion of anti-inflammatory cytokines [46,47,48,49,50] are reduced in obese AT [51,52,53]. Consequently, in obese AT, there is an increased cellular abundance of M1 macrophages, dendritic cells, CD4^+^ T cells, CD8^+^ T cells, natural killer cells and mast cells [47,48,54,55,56,57,58,59,60,61,62,63,64,65,66,67,68]. Both adipocytes and the various immune cell types present within the AT serve as cellular sources that produce a variety of inflammatory and immune cell chemotactic mediators that perpetuate immune cell infiltration into the AT and contribute to metabolic dysfunction [26,37,69]. Additionally, obese adipocytes have increased oxygen consumption and low oxygen pressure due to reduced blood supply, which together result in the formation of hypoxic environmental conditions within the AT [40,41,70], and can further perpetuate the production of inflammatory mediators and metabolic dysfunction [71,72,73]. Immune cells, in particular macrophages, encircle necrotic or dying adipocytes, particularly in hypoxic AT, forming crown-like structures (CLS), wherein a higher number of CLS is associated with worse inflammatory and metabolic outcomes including elevated C-reactive protein (CRP), dyslipidemia, hyperinsulinemia and IR as assessed using the homeostatic model assessment of insulin resistance (HOMA-IR) [74,75,76,77].

AT functions as an endocrine organ, wherein proteomic studies have identified over 600 adipokines, namely hormones, cytokines and chemokines released from AT, that contribute to chronic low-grade inflammation, local AT and systemic IR and metabolic dysfunction [37,71]. Both adipocytes and immune cell populations within AT serve as adipokine cellular sources, perpetuating a cycle in which increased chemotactic signals produced by AT promote the infiltration of innate and adaptive immune cell populations, increased secretion of inflammatory adipokines, and the development of IR and metabolic dysfunction [37,48,71,78,79,80]. As reviewed elsewhere [26,33,37], obesity is typically associated with increased expression of pro-inflammatory adipokines, including leptin, resistin, tumor necrosis factor alpha (TNF)-α, interleukin (IL)-6, monocyte chemoattractant protein (MCP)-1, visfatin, and chemerin, among others. In contrast, the secretion of anti-inflammatory adipokines, including, but not limited to, adiponectin, IL-10, adipsin, and omentin are reduced. Thus, higher levels of critical inflammatory adipokines are associated with increased obesity severity, IR and metabolic dysfunction. For example, circulating levels of leptin increase in obesity and are positively correlated with body fat mass [81,82,83]. Leptin has been shown to stimulate the production of inflammatory mediators, such as TNF-α and IL-6, in adipocytes and other cell types [84,85,86], promoting macrophage activation, proliferation and inflammatory adipokine production [84,85,87,88]. Other inflammatory stimuli, such as lipopolysaccharide (LPS) and inflammatory adipokines, can stimulate leptin secretion, creating a feed-forward loop that sustains inflammatory adipokine production [89,90,91]. Similarly, circulating TNF-α levels increase in obesity [92] and correlate with IR as assessed by HOMA-IR [93]. TNF-α impairs insulin signaling at the level of insulin receptor substrate (IRS) proteins [94], increases lipolysis and circulating free fatty acid (FFA) levels [95], and stimulates the expression of additional inflammatory mediators by activating the transcription factor nuclear factor kappa-light-chain-enhancer of activated B cells (NF-κB) [94]. This paracrine signaling loop results in the increased expression of other critical AT (namely adipocyte and macrophage)-derived mediators including MCP-1, TNF-α, and IL-6 and down-regulates the expression of adiponectin, that collectively maintains chronic low-grade AT inflammation and disrupts metabolic function [96,97,98]. In this connection, increased circulating IL-6 levels have been shown to predict the development of T2D [99] and are positively correlated with adiposity and circulating FFAs [100,101]. Importantly, IL-6 concentrations in AT interstitial fluid have been reported to be 100-fold higher versus circulating levels, highlighting the potential for IL-6 within the AT microenvironment to impact local tissue function in obesity [102]. AT IL-6 levels have been shown to be inversely correlated with adipocyte insulin-stimulated glucose uptake [100]. Responsiveness to IL-6 signaling via the expression of the IL-6 receptor (IL-6R) is increased in obesity and positively correlated with BMI and AT expression of other inflammatory mediators including TNF-α, IL-6 and MCP-1 [103], indicating that expression of IL-6 supports ongoing inflammatory mediator production. IL-6 signaling through the Janus Kinase (Jak)2/signal transducer and activator of transcription (STAT) 3 pathway has been shown to increase M1 macrophage-mediated AT inflammation and IR, wherein Jak2^−/−^ mice fed a high-fat diet exhibited improved insulin sensitivity, fewer AT CLS, reduced mRNA expression of IL-6, TNF-α, MCP-1 and IL-1β and M1 macrophage markers [104]. Similarly, STAT3 ablation has been shown to improve insulin sensitivity and glucose tolerance and suppress AT inflammation [105]. Furthermore, STAT3 ablation within macrophages has also been shown to improve obesity-associated insulin sensitivity and reduce the AT M1/M2 macrophage ratio [104]. Both AT and circulating levels of MCP-1 are increased in obesity [106,107,108]. MCP-1 is a potent chemokine that recruits monocytes to the AT and based on the inflammatory microenvironment in obese AT promotes their differentiation predominantly into M1 macrophages [77,109]. These macrophages become key cellular sources of additional inflammatory adipokines (such as TNF-α, IL-6, IL-1β and other chemokines including MCP-1) that further contribute to metabolic dysfunction [77]. Consistent with this, obese MCP-1-deficient mice exhibited improved metabolic function, reduced adiposity, decreased AT macrophage accumulation, increased serum levels of adiponectin and/or reduced AT expression of TNF-α [106,110]. Resistin levels are also increased in obesity [111,112], and circulating resistin levels positively correlate with IR (assessed by HOMA-IR) in humans with obesity [93]. Furthermore, resistin has been shown to sustain AT inflammation by activating NFκB and driving the expression of inflammatory adipokines including MCP-1 and IL-6, amongst others [113]. Furthermore, resistin has been shown to play a role in the suppression of insulin-mediated signaling in adipocytes by activating suppressor of cytokine signaling 3 (SOCS3) [114], although circulating resistin levels in humans only weakly correlate with body fat or BMI [115]. Conversely, beneficial effects of the anti-inflammatory, lipid-oxidation-promoting and insulin-sensitizing adipokine, adiponectin [88,90,116], whose circulating levels have been shown to be negatively correlated with IR (assessed via HOMA-IR) [93] are attenuated in obesity [117,118]. Collectively, these alterations reflect a shift toward an inflammatory adipokine profile in obese AT that sustains chronic low-grade inflammation and connects to metabolic dysfunction and increased susceptibility to obesity-associated comorbidities [12,13,14].

## 3. LC n-3 PUFA and Adiposity

Pre-clinical research has demonstrated the beneficial effects of LC n-3 PUFA, consisting of attenuating fat accumulation, improving adipocyte function, and promoting favorable remodeling of AT through metabolic, anti-inflammatory, and thermogenic mechanisms. In cell culture, adipocyte treatment with EPA has been associated with increased EPA incorporation into lipid droplets, decreased lipid droplet size, and decreased oxidative stress, suggesting a role for fatty acid composition in regulating adipocyte hypertrophy [119]. In animal models of obesity, dietary EPA and DHA from fish oil have consistently been associated with decreased adiposity and improved lipid metabolism. Studies in high-fat diet (HFD)-induced obese mice have reported that EPA and DHA mitigate the severity of obesity by decreasing body weight, reducing adiposity and preventing AT accumulation, resulting in decreased AT weight and adipocyte size, while promoting AT remodeling toward a non-obese phenotype [120,121,122,123,124,125,126,127]. Additionally, EPA and DHA have been associated with decreased adipose cell hypertrophy [127,128,129,130], which may contribute to reduced fat mass [120,121] and reduced adipocyte hyperplasia [120,127]. Mechanistically, reductions in adiposity, hypertrophy, and hyperplasia may be due to EPA- and DHA-associated improvements in mitochondrial function and restored homeostasis in the tricarboxylic acid cycle (TCA) cycle; however, more research is needed to further explore these mechanisms.

EPA and DHA have also been shown to promote the browning of WAT in rodent models of obesity [128,131,132]. This may occur via the upregulation of beige adipocyte gene expression markers (uncoupling protein 1; UCP1 and protein kinase B (pAKT/AKT) or by upregulating the production of inflammation pro-resolving mediators derived from LC n-3 PUFA (maresins and protectins) in brown AT, upregulating brown AT activity and/or improving thermogenic signaling [131]. DHA may facilitate the browning of WAT into thermogenically active beige AT and enhance brown AT thermogenesis by increasing oxidative metabolism and upregulating the expression of thermogenic genes, which contribute to enhanced metabolic plasticity and energy expenditure [132,133]. Specifically, DHA has been shown to promote the remodeling of WAT by reducing adipocyte size, suppressing lipogenesis, and enhancing beige adipocyte gene expression [132]. Furthermore, DHA has been associated with the increased expression of UCP1 and PR domain containing 16 (PRDM16), which contribute to AT thermogenesis and the regulation of brown/beige adipocyte differentiation [132]. Similarly, DHA has been shown to reverse the suppression of browning markers in HFD-induced obese mice by improving mitochondrial function [upregulation of peroxisome proliferator-activated receptor gamma coactivator 1 alpha (PGC1α)] and restoring thermogenic capacity by upregulating genes involved in mitochondrial biogenesis [UCP1 and cell death-inducing DFFA-like effector A (CIDEA)] [133]. Conversely, findings on the effects of ALA on adiposity are mixed. Some studies suggest that ALA from flaxseed oil does not prevent fat accumulation and may not mitigate adiposity in HFD-induced obesity in rodents [123,134]. While dietary supplementation with chia seeds (rich in ALA) has been shown to reduce visceral adiposity [135] in rodent models of obesity, these differences may be linked to the source of ALA utilized (i.e., flax oil extracts versus chia seeds), or the form of dietary incorporation (e.g., whole food supplementation from chia seeds versus oil extracts) that may influence the reported effects.

In human studies, the effects of LC n-3 PUFA on aspects of body weight and adiposity are conflicting. Fish oil (rich in EPA and DHA) supplementation in individuals with overweight following a weight-loss diet was shown to reduce abdominal fat mass [136], whereas other research has shown no difference between fish oil supplementation and placebo [137]. It is hypothesized that changes in weight and adiposity associated with fish oil supplementation may occur by various mechanisms, including appetite suppression, improvements in blood circulation, and changes in fatty acid metabolism [138]. In studies where EPA and DHA were not associated with significant changes in body weight, BMI, or body composition, other beneficial effects on elements of the obese phenotype have nevertheless been reported [139,140]. Conversely, evidence suggests that ALA has no effect on body composition in adults with overweight, further supporting the interpretation that ALA may not exert the same effects on elements of the obese phenotype compared to the LC n-3 PUFA EPA and DHA [141]. Human clinical data are limited; however, a recent meta-analysis of seven studies comparing the effects of LC n-3 PUFA versus placebo showed a significant reduction in waist circumference, despite no significant difference in BMI or weight loss [142]. Of the limited number of studies included in the meta-analysis, there was variability in the findings that could be attributed to, at least in part, the dosage of LC n-3 PUFA used (i.e., 2–6 g), the range of treatment duration (i.e., 3–24 weeks), and the incorporation (or lack thereof) of lifestyle adjustments during the trial duration, including exercise and restricted caloric intake to induce weight loss [142]. Importantly, there are other critical obesity-related outcomes beyond anthropometric measurements that have been shown to be affected by LC n-3 PUFA human clinical trials; however, there is variability in the outcomes across studies for both anthropometric and biochemical outcomes, as well as metabolic and inflammatory outcomes, as shown in Table 1 and discussed further in Section 4. Sources of variability include the outcome measures evaluated versus those that are relevant but not evaluated in a given study, and heterogenous findings within a single outcome across multiple studies. These differences are likely attributable to discrepancies in the various study designs including LC n-3 PUFA dose, duration of intervention, sample size and subject characteristics that are not stratified for age, gender, BMI status (i.e., overweight versus obesity), metabolic status (i.e., study participants with or without concurrent insulin resistance, dyslipidemia and/or other cardiometabolic risk factors) and inflammatory status (i.e., study participants with obesity wherein inflammatory complications or elevated biomarker expression has not yet developed). Therefore, studies that characterize their participants’ metabolic and inflammatory status in addition to obesity-associated anthropometric outcome measures prior to intervention with LC n-3 PUFA [137,139,143,144,145,146] may help to address some of the heterogenous findings in human intervention studies. Collectively, the findings summarized in Table 1 and discussed further in Section 4 support the potential role of LC n-3 PUFA supplementation for obesity prevention and management; however, further study is required, as mechanistic insights into the effects of LC n-3 PUFA in obesity are more concentrated in pre-clinical studies.

## 4. Effects of n-3 PUFA on AT Inflammation and Metabolic Dysfunction

LC n-3 PUFA have been shown to exert anti-inflammatory effects through increased production of EPA- and DHA-derived oxylipids and pro-resolvins in WAT [127] and plasma [156]. Additionally, a more substantive anti-inflammatory mechanism of action is reflected through the effects of LC n-3 PUFA on the obesity-associated adipokine profile, including increased expression of anti-inflammatory and insulin-sensitizing adipokines such as adiponectin, which was concomitant with reduced production of several inflammatory adipokines across various obesity models and experimental systems.

A critical effect of LC n-3 PUFA on obese AT function is the increased secretion of adiponectin, which exerts both anti-inflammatory and insulin-sensitizing effects that are typically lost in obesity [93,117,118]. In rodent models of adiponectin deficiency, IR develops, whereas adiponectin overexpression has been shown to improve insulin sensitivity and glucose tolerance [157]. Moreover, when adiponectin levels are restored in obese mice, glucose and lipid metabolism are improved along with decreased circulating inflammatory mediator levels and macrophage infiltration into the AT [158], thereby improving the AT cellular composition. LC n-3 PUFAs have been shown to increase adipokine expression or production in adipocytes and/or primary AT [159,160,161,162,163,164,165] and circulating levels of adiponectin are increased by LC n-3 PUFA in human subjects with obesity [137,150,160,164,166,167,168,169,170,171] and in rodent obesity models [127,161,162,163,164,172,173,174,175]. Additionally, there are reports showing no effect of LC n-3 PUFA on circulating adiponectin levels [140,145,155,176]. Obese AT-derived inflammatory mediators have been shown to down-regulate adiponectin expression [177,178,179,180], thereby demonstrating how chronic inflammation contributes to AT dysfunction; however, the effects of LC n-3 PUFA on reducing inflammatory adipokine expression in obese AT can disrupt this cycle. Leptin is a critical obesity-associated adipokine, as leptin signaling results in the expression and secretion of inflammatory mediators (such as TNF-α and IL-6) from multiple AT cellular sources including adipocytes, macrophages, and T cells [84,85,181]; therefore, through this mechanism, leptin can sustain chronic low-grade inflammation within obese AT. LC n-3 PUFAs have been shown to reduce AT expression and/or circulating leptin levels in primary AT or in obese rodent models [120,126,127,160,161,175,182,183] and in humans living with overweight or obesity [137,151,167,168,184,185]. However, there are also reports showing no effect of LC n-3 PUFA on circulating leptin levels in human clinical trials [140,145,155,160,176], although other elements of the obese phenotype (apart from leptin levels) were improved in these studies.

**Figure 2:**
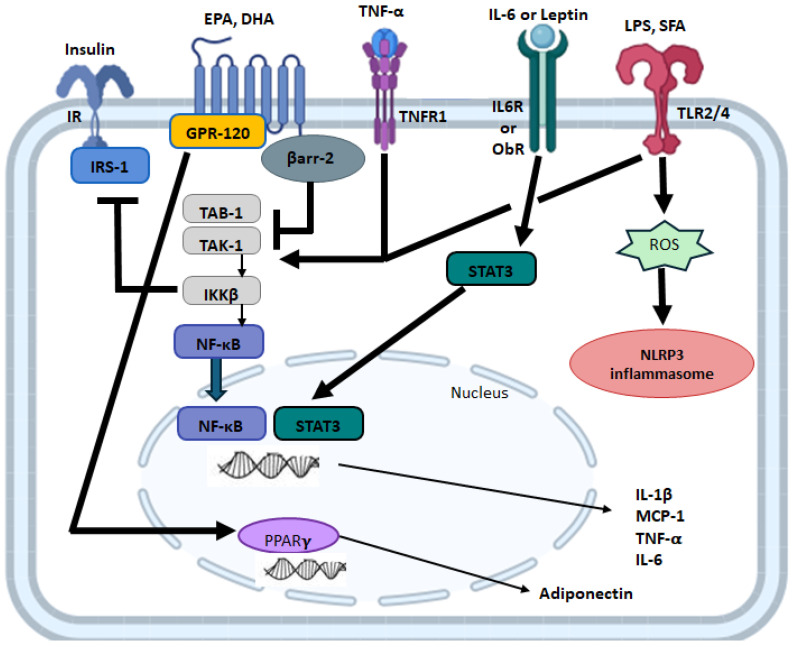
Simplified select signaling mechanisms in obese AT leading to adipokine production. LC n-3 PUFAs stimulate GPR120, which promotes the association between β-arrestin 2 (βarr2) and GPR120. βarr2 binds to transforming growth factor-β-activated kinase (TAK)1 binding protein (TAB)1, which inhibits TAK1/TAB1 binding and subsequent NFκB activation. TNF-α binds to the TNF Receptor 1 (TNFR), which promotes TAK1/TAB1 binding and NFκB activation. LPS and SFA stimulate TLR2 and TLR4, which promote TAK1/TAB1 binding and NFκB activation. EPA and DHA activation of PPARγ is through both GPR120-dependent and independent mechanisms. IL-6 binds to the IL-6 receptor (IL6R) and leptin binds to the leptin receptor (ObR), leading to STAT3 activation. TLR2- or TLR4-induced reactive oxygen species (ROS) accumulation leads to the activation of the inflammasome.

AT expression and secretion of inflammatory adipokines (namely cytokines and chemokines) are also reduced by LC n-3 PUFA, with stronger evidence reported in animal and cell culture studies. In human studies with overweight or obesity, the effects of LC n-3 PUFA supplementation show conflicting findings, which may be attributable to differences in study designs, dose of LC n-3 PUFA supplementation (e.g., ranging from 0.6 to 6.0 g/day of EPA + DHA), the source of LC n-3 PUFA (namely dietary sources versus supplements), the duration of intervention and differences in the endpoints evaluated across studies. Differences in endpoints evaluated (in addition to adiponectin and leptin) include inflammatory mediators such as TNFα, IL-6, MCP-1 (commonly included in pre-clinical studies) or broader clinically relevant inflammatory biomarkers such as CRP, which has been shown to be predictive of T2D development [186,187]. Importantly, some clinical trials have shown that LC n-3 PUFA supplementation in humans with obesity reduce circulating levels of TNF-α, IL-6, MCP-1 and/or CRP [140,145,146,151,171,188,189]. Conversely, some intervention trials involving humans with obesity have reported no relationship between LC n-3 PUFA intake and circulating levels of various inflammatory mediators [137,140,145,150,152,190,191,192,193]; however, other aspects of the obese phenotype and/or risk factors for obesity-associated comorbidities were beneficially affected in these studies. Evidence reported in cell culture and animal studies have demonstrated a reproducible effect of LC n-3 PUFA in reducing the expression and/or secretion of inflammatory adipokines (namely cytokines and chemokines). In both in vitro studies (utilizing adipocyte cell lines alone or in co-culture or obese AT explants) and animal obesity models, LC n-3 PUFAs have been shown to reduce AT expression and/or the secretion of TNF-α [127,175,194,195,196,197], IL-6 [126,127,194,195,197,198,199,200,201,202], IL-1β and/or inflammasome activation [197,199,200,201,203] and MCP-1 [126,127,129,162,174,175,194,195,196,197,198,199,200,201,203,204]. Moreover, LC n-3 PUFAs have been shown to reduce the activation of the inflammatory transcription factors NFκB [129,195,196,197,198,199,200,203,205] and STAT3 [196,199], that drive the expression of the aforementioned inflammatory mediators. Additionally, LC n-3 PUFAs have been shown to increase the expression of the anti-inflammatory cytokine IL-10 [161,174,201,205]. Mechanistically, the effects of LC n-3 PUFA in reducing inflammatory mediator production are attributed, at least in part, to the inhibition of Toll-like receptor (TLR) activation that leads to decreased NFκB activation, increased peroxisome proliferator-activated receptors’ gamma (PPARγ) activation, G-protein coupled receptor 120 (GPR120) signaling or down-regulation of NOD-like receptor family pyrin domain containing three (NLRP3) inflammasome components [140,197,201,204,206,207,208,209,210,211,212,213], and signaling mechanisms that underlie obesity-associated inflammatory mediator production (summarized in Figure 2), although further study is required. Collectively, these data demonstrate an anti-inflammatory and anti-chemotactic shift in the obese adipokine profile in response to LC n-3 PUFA that can contribute to reducing the chronic low-grade inflammation and metabolic dysfunction that characterizes obese AT.

Reduced chemokine expression has also been found with LC n-3 PUFA, including MCP-1 [126,129,174,175,194,195,196,197,198,199,200,201,203], MCP-3, macrophage inflammatory protein (MIP)-1α, MIP-1β and, regulated on activation, normal T expressed and secreted (RANTES) [196,197,199,200], which are important for reducing immune cell infiltration, in particular macrophages, into obese AT. As such, LC n-3 PUFAs have been shown to decrease the number of CLS within obese AT [127,130], and decrease monocyte chemotaxis and polarization to inflammatory M1 macrophages in AT, and/or promote the polarization of macrophages toward the M2 polarization state [129,145,160,162,172,174,182,196,201,214,215,216,217,218,219]. Furthermore, studies have demonstrated attenuated monocyte or macrophage inflammatory mediator mRNA expression or secretion (such as TNF-α, IL-6, IL-1β), increased IL-10 secretion, reduced NFκB activation and/or improved insulin sensitivity [207,214,218,220,221].

Thus, cross-talk between adipocytes and immune cell populations, primarily CD8^+^ or CD4^+^ T cells and macrophages, has been shown to drive the production of inflammatory mediators in co-culture studies, which is antagonized with the addition of LC n-3 PUFA. In CD8^+^ T cell/adipocyte co-cultures that recapitulate the cellular ratio in obese AT, the resultant secretory profile was both anti-inflammatory and anti-chemotactically evidenced by decreased levels of TNF-α, IL-6, MCP-1, MCP-3 and MIP-1β [196,197,222]. Macrophages treated with the conditioned media from the LC n-3 PUFA-treated CD8^+^ T cell/adipocyte co-culture media exhibited reduced macrophage chemotaxis [196,222] and a shift in the expression of macrophage polarization markers that reflected increased M2 and decreased M1 polarization [197]. A similar experimental approach with CD4^+^ T cell/adipocyte co-cultures that recapitulate the cellular ratio in obese AT demonstrated that LC n-3 PUFAs promote T cell differentiation into the non-inflammatory Th2 subset and reduced Th1 differentiation combined with the reduced secretion of inflammatory and macrophage chemotactic mediators including IL-1β, IL-6, MCP-1, MCP-3 and MIP-1α [199,210]. These changes within T cell populations are highly relevant, as research has shown that T cells infiltrate into obese AT prior to monocyte chemotaxis and M1 polarization and metabolic dysfunction [48]. Finally, in macrophage/adipocyte co-culture, the addition of LC n-3 PUFA has been shown to reduce inflammatory mediator production including (TNF-α, IL-6, RANTES, MCP-1, and MCP-3), in part, via mechanisms that include reducing NLRP3 inflammasome expression and cellular caspase-1 activity [198,200]. Collectively, these studies indicate that LC n-3 PUFA can disrupt the critical cross-talk between adipocytes and T cells that leads to the recruitment of macrophages, their inflammatory subset polarization, low-grade inflammatory mediator production and the onset of metabolic dysfunction.

Both obesity and obesity-associated inflammation have been linked with the development of IR and T2D. Studies have consistently shown that individuals with glucose intolerance and IR display elevated levels of circulating CRP, IL-6, and TNF-α, even in the pre-diabetic state [223,224,225,226]. Elevated CRP levels have been associated with risk of developing T2D [186,187,223,227,228,229,230,231]. Furthermore, elevated TNF-α has been associated with increased IR and elevated levels of plasma FFA and triglycerides, which are known risk factors for developing T2D [232].

In the case of T2D, the anti-inflammatory effects of LC n-3 PUFA have been shown to impact cell membrane function and insulin signaling, as reviewed elsewhere [233]. In cell culture studies, adipocytes treated with DHA (50 to 100 µM) have been shown to increase glucose transport, which contributed to insulin sensitization [201], glucose transporter type 4 (GLUT4) translocation and increased glucose uptake [214]. The beneficial effects of LC n-3 PUFA on metabolic outcomes and insulin sensitivity have also been reported in animal obesity models, wherein dietary supplementation of fish oil has been shown to improve insulin sensitivity in adipocytes and skeletal muscle [120,163,234,235,236]. A more recent study reported that this increased insulin sensitivity was associated with the mammalian target of rapamycin complex 2 (mTORC2) activation, which may provide insights on the mechanism underlying the beneficial effects of LC n-3 PUFA on insulin sensitivity and metabolic outcomes [236]. The effects of LC n-3 PUFA on metabolic outcomes in human studies are mixed. Some evidence suggests supplementation with high-dose EPA (1.3 g/day) and DHA (2.9 g/day) improves insulin sensitivity in females with overweight and obesity [146]. These findings are also supported by more recent studies, which have reported that varying doses of EPA and DHA are associated with decreased insulin concentrations and/or HOMA-IR in males and females with overweight and obesity [137,140,146,154,155,237]. Conversely, other studies have reported no beneficial effects of LC n-3 PUFA on circulating insulin levels [143,154,155] or insulin sensitivity [143,145], including a high-dose DHA supplementation (3.9 g/day); however, this may be due to the participants’ insulin sensitivity state [153], and may also suggest non-linear dose relationships and highlight metabolic complexity. Similarly, human clinical interventions with LC n-3 PUFA have shown variable findings with respect to fasting blood glucose levels, which have been shown to decrease [151], increase [150] and be unaffected [143,155] by LC n-3 PUFA. Similarly, biomarkers of obesity and insulin resistance-associated dyslipidemia [238] have been shown to improve in response to LC n-3 PUFA supplementation in human clinical trials, including decreased plasma triacylglycerides [137,144,145,146,151,152], very-low-density lipoproteins (VLDLs) [143], low-density lipoprotein (LDL) cholesterol and total cholesterol [151], while also increasing high-density lipoprotein (HDL) cholesterol [144]. Importantly, conflicting findings have been reported, wherein LC n-3 PUFAs have been shown to have no effect on circulating triacylglycerides [140,150,155], LDL cholesterol [143,145,150,155], HDL cholesterol [143,145,150,155], and total cholesterol [140,143,145,150,155]. These heterogenous findings in the human clinical data are summarized in Table 1.

**Figure 3:**
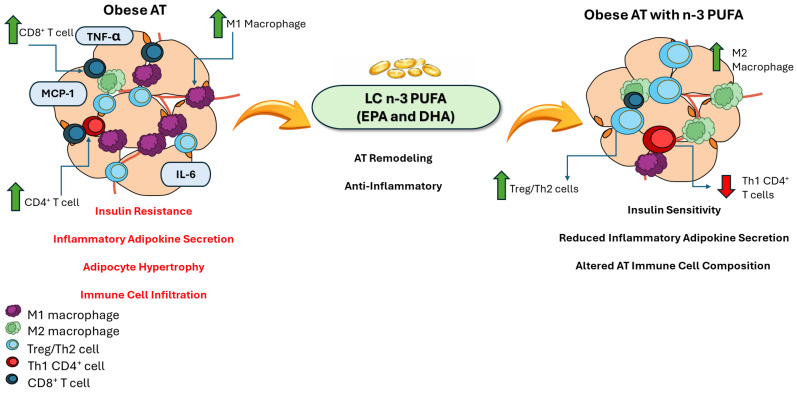
Summary of the effects of n-3 PUFA on obese AT function.

Collectively, these findings suggest a role for LC n-3 PUFA in disrupting the inflammatory cascade that occurs in obesity, that could contribute to decreased production of pro-inflammatory mediators and obesity-associated chronic inflammation and metabolic dysfunction (Figure 3); however, future studies are needed to assess these mechanisms of action within AT-derived cellular populations (namely both immune cell populations and adipocytes).

## 5. Obese Skeletal Muscle

### 5.1. Obesity-Associated Changes in Skeletal Muscle and Cross-Talk with AT

As previously mentioned, obesity can lead to the development of IR and subsequently T2D. Skeletal muscle is the primary site for insulin-simulated glucose uptake and utilization; therefore, it is the main organ responsible for maintaining whole-body glucose homeostasis [239,240]. In brief, the insulin signaling pathway consists of a series of steps that are initiated by the binding of insulin and insulin-like growth factors (IGFs) to their respective receptors on the skeletal muscle membrane, followed by the binding of the insulin receptor to its respective insulin receptor substrate (IRS) upon activation [241]. Briefly, IRS-1 undergoes tyrosine phosphorylation, which recruits phosphoinositide 3-kinase (PI3K) and phosphorylates PIP2 to PIP3, leading to Akt phosphorylation and subsequent promotion of GLUT4 trafficking [241,242].

**Figure 4:**
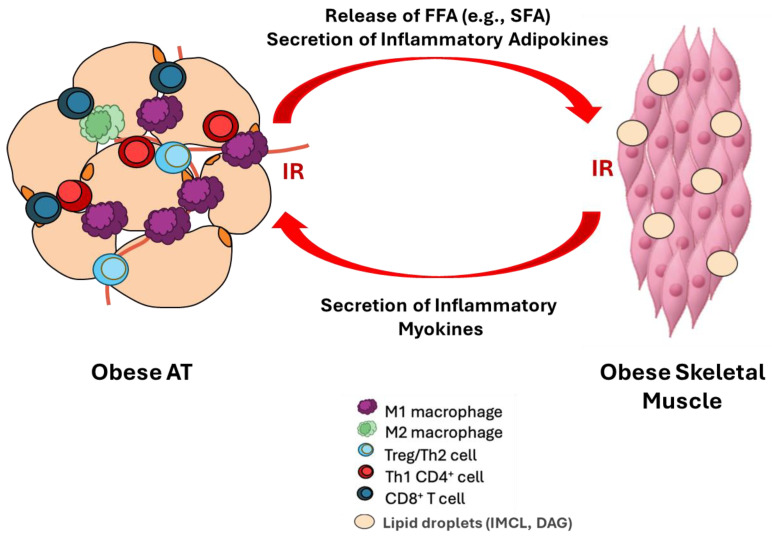
Inter-tissue cross-talk between obese AT and skeletal muscle promoting IR.

In obesity, skeletal muscle IR develops and can lead to systemic IR [240,243], through multiple interacting mechanisms. For instance, impaired triglyceride storage capacity in the AT can result in increased circulating FFA and ectopic fat deposition in peripheral tissues, such as skeletal muscle, wherein lipid accumulation can impair tissue function and contribute to the development of IR [45]. Therefore, signals originating from AT including increased circulating FFA, in particular free saturated fatty acids (SFAs) and inflammatory adipokines, can both interfere with insulin signaling in skeletal muscle to promote local tissue metabolic dysfunction and contribute to the development of whole-body IR [34,244,245,246,247,248]. This forms the basis of inter-tissue cross-talk between obese AT and the skeletal muscle (Figure 4).

A consequence of excess circulating SFA (from AT) is the development of intramyocellular lipids (IMCLs) due to ectopic lipid accumulation [249]. The accumulation of ectopic fat in skeletal muscle also contributes to the production of diacylglycerols (DAGs), particularly through IMCL, triggering increased protein kinase C (PKC)-θ which results in serine phosphorylation of IRS-1, thereby impairing insulin signaling [249,250]. Additionally, ceramide production is increased in obese skeletal muscle and results in elevated protein kinase C (PKC)-zeta levels, leading to a reduction in Akt phosphorylation by suppressing PIP3 binding, contributing to already worsened insulin signaling capabilities [40]. Mitochondrial reactive oxygen species (ROS) emission rates have been shown to increase [251], whereas ADP-stimulated mitochondrial oxidative capacity has been shown to decrease in obese skeletal muscle [252]. Elevated circulating levels of AT-derived adipokines can also affect skeletal muscle function, wherein MCP-1 has been shown to impair insulin signaling through impaired Akt and glycogen synthase kinase (GSK)-3 α/β phosphorylation and GLUT4 translocation, thereby compromising insulin-stimulated glucose uptake in skeletal muscle [253]. Similarly, TNF-α and IL-1β have been shown to impair IRS-1 phosphorylation through the activation of PKC, c-Jun N-terminal kinase (JNK), and IKK/NF-κB pathways [254,255], wherein they form a positive feedback loop that promotes further local tissue production of TNF-α, IL-1β and IL-6, thereby perpetuating skeletal muscle production of inflammatory mediators that can contribute to impaired IRS function and metabolic dysfunction [40,256]. Resistin has been shown to exert pro-inflammatory effects and to increase lipid accumulation in the muscle, primarily through NF-κB and mitogen-activated protein kinase (MAPK) activation [245,257]. Furthermore, obesity can result in the increase in fibro-adipogenic progenitors (FAPs) in skeletal muscle [245,257]. Although FAPs play important structural and metabolic roles in the skeletal muscle, under obese conditions, they undergo a phenotype-shift that results in deleterious effects [245]. In the skeletal muscle, the FAPs contribute to intramyocellular lipid accumulation, leading to impaired insulin signaling and glucose uptake [245,257]. Similarly to AT, the obese skeletal muscle is characterized by the infiltration and accumulation of immune cells, particularly M1 macrophages and T cells, which serve as cellular sources of inflammatory mediators within the skeletal muscle and contribute to local inflammation and IR through immune cell–myocyte cross-talk [255,258,259,260,261,262,263], although reduced mRNA expression of macrophage surface markers in obese skeletal muscle has been reported [122]. Thus, there is cross-talk between obese AT and skeletal muscle, wherein the endocrine communication signals from AT include FFA (such as SFA) and secreted inflammatory adipokines that impact skeletal muscle function and promote IR [34,244,245,246,247,248]. This communication between tissues (i.e., cross-talk) can be bi-directional with adipokine signals impacting skeletal muscle function and myokine signals impacting AT function, as shown in Figure 4 and reviewed elsewhere [245,264,265]. Given that AT and skeletal muscle are major regulators of systemic metabolism, it is important to understand how the cross-talk between these tissues (predominantly driven by AT-derived FFA and adipokines) can influence skeletal muscle function and collectively contribute to obesity-associated whole-body metabolism.

### 5.2. Effect of LC n-3 PUFA on Obese Skeletal Muscle Function

**Figure 5:**
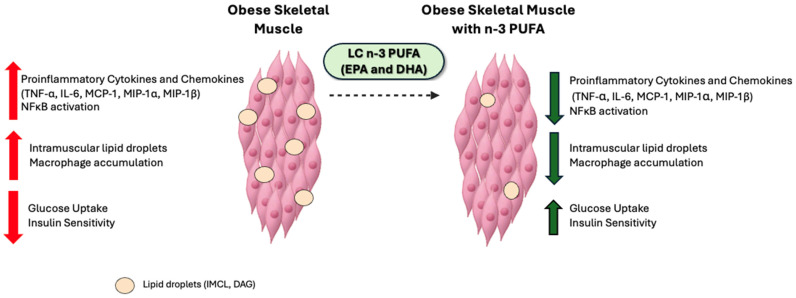
Summary of effects of LC n-3 PUFA on obese skeletal muscle function.

Obesity negatively impacts skeletal muscle function and creates an inflammatory environment within that tissue that often results in impaired insulin signaling and the development of IR. Therefore, LC n-3 PUFAs are increasingly studied as a potential strategy to reduce the inflammatory environment within the skeletal muscle (along with AT), to subsequently improve insulin signaling (Figure 5). While several studies have demonstrated beneficial effects of LC n-3 PUFA on obese skeletal muscle function, conflicting evidence remains. These inconsistencies may be due to differences in experimental design, including variations in LC n-3 PUFA dose and duration of exposure, as well as the use of different skeletal muscle cell culture and co-culture models.

Glucose uptake following a high dose of palmitic acid, which has been shown to induce IR, was improved in L6 myotubes treated with either 100 μM EPA or 100 μM DHA [266]. Similarly, in a macrophage–myocyte (RAW 264.7 macrophages + L6 myotubes) co-culture model with LPS stimulation (to recapitulate obese skeletal muscle environmental conditions), DHA (100 μM) was shown to improve glucose uptake and also reduce secretion of pro-inflammatory cytokines (TNF-α, IL-6, MCP-1) and chemokines (MIP-1α and MIP-1β) [267]. In this connection, primary human myoblasts treated with EPA (100 μM) following a high dose of palmitic acid to induce metabolic dysfunction displayed lower IL-6 mRNA expression and NFκB activation with higher expression of the inhibitor IκBA [268]. Therefore, the anti-inflammatory effect observed with LC n-3 PUFA treatment may be attributed to reduced activation of JNK and MAPK, and NFκB [268,269]. Conversely, a lower dose of EPA (50 μM) was shown to improve basal (i.e., non-insulin-stimulated) glucose uptake in L6 myotubes, whereas DHA (50 μM) had no effect, and neither LC n-3 PUFA affected insulin-stimulated glucose uptake [270]. Additionally, a higher dose (400 μM) of DHA was shown to have no effect on both basal and insulin-stimulated glucose uptake in L6 myotubes [271].

Macrophage accumulation within the skeletal muscle, as well as the ensuing macrophage–myocyte cross-talk, has been shown to increase the production of inflammatory mediators (i.e., myokines), leading to obesity-associated metabolic dysfunction [260]. In vitro, macrophages treated with DHA (100 µM) + LPS to recapitulate obese-circulating endotoxin levels exhibited reduced gene expression of TNF-α and increased gene expression of the anti-inflammatory cytokines IL-10 and transforming growth factor (TGF)-β; in addition to decreasing the secretion of IL-6 and MCP-1 [267]. Furthermore, when DHA-treated macrophage-conditioned media was added to L6 myotube cultures, the secretion of skeletal muscle cell-derived cytokines (TNF-α and IL-6) and chemokines (MCP-1, MIP-1α and MIP-1β) were reduced and insulin-stimulated glucose uptake was increased [267]. In animal obesity models, macrophage infiltration into skeletal muscle does not accumulate surrounding the myofibrils but instead increase within intermuscular fat depots [175]. Furthermore, HFD-associated increases in CD11c^+^ immune cell content within the skeletal muscle are associated with the increased expression of TNF-α and insulin resistance [272]. Separately, increased HFD-associated skeletal muscle gene expression of the macrophage markers F4/80 and CD11c coincided with increased inflammatory mediator expression (TNF-α and IL-6), increased intramuscular triglyceride, DAG and cholesterol ester content and changes in the fatty acid composition of skeletal muscle, resulting in lower levels of EPA and DHA [273]. Interestingly, LC n-3 PUFA supplementation to a HFD has been shown to reduce both total lipid content, TNF-α expression and galectin3 (Gal3)-expressing macrophages within the skeletal muscle [175]. With respect to the decreased intramuscular lipid levels, saturated fatty acid-induced increases in ceramides and DAG in C2C12 myotubes were decreased by both plant-derived (ALA) and marine-derived (EPA and DHA) n-3 PUFA [274]. Similarly, in obese animal studies, despite no effect on intramuscular triglyceride levels, LC n-3 PUFAs have been shown to reduce obese skeletal muscle accumulation of both ceramides and their immediate precursors long-chain acyl-CoAs, while also improving glucose tolerance [275]. Although other studies have shown no effect of LC n-3 PUFA supplementation on obese skeletal muscle triglyceride and ceramide levels in HFD-induced obesity models, it is important to highlight that other critical aspects of the obese phenotype were improved, including reduced whole-body IR and reductions in adipocyte size and the number of CLS within WAT [130].

In rodent studies, inclusion of LC n-3 PUFA in a HFD has been shown to improve insulin sensitivity and/or reduce HOMA-IR [161,175,276,277,278,279], although findings are conflicting including those reporting no improvement in insulin sensitivity [280,281]. Additionally, LC n-3 PUFA supplementation in a HFD has been shown to increase muscle PPARγ expression [276], which not only influences fatty acid metabolism but also exerts beneficial effects by reducing the expression of inflammatory mediators [282]. Interestingly, the plant-derived LC n-3 PUFA ALA has also been shown to improve obesity-associated impairments in glucose homeostasis in response to either an oral [279] or intraperitoneal [283] glucose tolerance test. In human clinical studies, daily supplementation of 2.7 g of LC n-3 PUFA (as DHA and EPA combined) over three months to males and females with T2D was shown to reduce HOMA-IR [154]. Similarly, a lower daily supplementation level of 1.2 g LC n-3 PUFA (930 mg EPA and 290 mg DHA) for three months was shown to improve glucose tolerance and insulin sensitivity only in adolescent females with obesity, whereas there was no effect in males [155], highlighting the importance of investigating sex-specific effects of LC n-3 PUFA. Collectively, these findings suggest that LC n-3 PUFA supplementation may mitigate inflammatory processes in the obese skeletal muscle, which may attenuate insulin signaling defects.

## 6. Conclusions

This review explored the effects of LC n-3 PUFA on obese AT and skeletal muscle function, which mechanistically disrupt inflammatory mediator (adipokines in AT and myokines in skeletal muscle) production that functionally connects obesity-associated low-grade chronic inflammation to metabolic dysfunction through multiple concurrent mechanisms, as summarized in Table 2. These effects can not only improve local (i.e., within tissue) function in obesity but can also attenuate the severity of the systemic obese phenotype by reducing inter-tissue cross-talk between AT and the skeletal muscle (Figure 4). Pre-clinical data (i.e., in vitro and animal studies) provide the framework for critical signaling pathways and cellular-level changes within both AT and skeletal muscle that underlies obesity-associated changes in adipokine and myokine production and contributes to metabolic dysfunction (e.g., IR); the data are also supported by the findings from human intervention studies, although there are heterogenous findings (as discussed herein and summarized in Table 1).

Studies ranging from pre-clinical (both cell culture and rodent models) to human clinical trials provide evidence for the role of LC n-3 PUFA, specifically EPA and DHA, in attenuating critical aspects of the obese phenotype. Despite promising pre-clinical and observational evidence, clinical trials have yielded heterogeneous outcomes, partly due to interindividual variability, disparities in the dosage and duration of intervention, use of dietary or supplement forms of LC n-3 PUFA, and diversity in the inflammatory and metabolic endpoints evaluated. Overall, while LC n-3 PUFAs represent a biologically plausible adjunctive strategy to mitigate obesity severity, further rigorously controlled human studies that comprehensively assess a range of inflammatory and metabolic endpoints are needed to clarify the optimal supplementation level to fully elucidate their therapeutic potential.

## Figures and Tables

**Table 1:** Summary of LC n-3 PUFA intervention studies in humans with obesity ^1^.

Study Population [Reference]	Intervention	Effects of n-3 PUFA
Males and females (aged 30–60 years old) with overweight or obesity on a weight-loss diet program for 12 weeks [136]	**n-3 PUFA Group** (*n* = 20): 1020 mg n-3 PUFA/day (consisting of 580 mg EPA, 390 mg DHA, and 50 mg other n-3 PUFA) for 12 weeks, in addition to 150 g salmon consumed twice weekly **Control Group** (*n* = 20): 150 g salmon consumed twice weekly, 12 weeks	n-3 PUFA Group: ↓ Abdominal fat mass ↓ Abdominal fat percentage
Males (aged 58 ± 8 years old) with obesity and dyslipidemia [143]	**n-3 PUFA Group** (*n* = 12): 4 g/d fish oil capsule, consisting of 45% EPA and 39% DHA as ethyl esters, for 6 weeks **Placebo Group** (*n* = 12): corn oil placebo capsule, for 6 weeks	n-3 PUFA Group: ↓ plasma triacylglycerides and VLDL triacylglycerides versus placebo ↓ VLDL production versus placebo No change in plasma total cholesterol, LDL or HDL cholesterol, glucose, insulin or HOMA-IR score from baseline
Females with overweight or obesity and hyperinsulinemia [137]	Weight-loss program (to achieve 10% weight loss in 12 weeks, followed by 12 weeks of a weight maintenance phase **Weight Loss + n-3 PUFA/fish oil group** (**WLFO**; *n* = 35): 1.3 g EPA + 2.9 g DHA per day (for 24 weeks) **Weight Loss + Placebo oil group** (**WLPO**; *n* = 32): 2.8 g linoleic acid + 1.4 g oleic acid per day (for 24 weeks) **Control group** (*n* = 26): no weight loss and placebo oil (2.8 g linoleic acid + 1.4 g oleic acid per day; for 24 weeks)	Anthropometric outcomes WLFO versus Control: ↓ weight, waist circumference, fat mass and abdominal fat (at 12 and 24 weeks of intervention). No significant difference in these parameters between WLFO and WLPO groups. Clinical and plasma biochemical outcomes WLFO versus Control (after 24 weeks of intervention): ↑ Insulin sensitivity ↓ Triacylglycerides ↓ Leptin ↑ Adiponectin WLFO versus WLPO (after 24 weeks): ↓ Triacylglycerides
Males and females with obesity but not diagnosed metabolic or inflammatory complications (aged 18–65 years old) [139]	**Fish oil group** (*n* = 22): 3 g fish oil/day (providing 1.1 g EPA + 0.8 g DHA) for 12 weeks **Corn oil group** (*n* = 21): 3 g corn oil/day (providing 1.65 g linoleic acid + 0.81 g oleic acid) for 12 weeks	Fish oil and corn oil groups with obesity exhibited no difference in outcomes compared to non-obese fish oil- and corn oil-supplemented groups for BMI, % body fat, body fat mass, waist and hip circumference and circulating levels of total cholesterol, LDL cholesterol and HOMA-IR. Fish oil and corn oil groups were the “normal range” for circulating triacylglycerides, non-esterified fatty acids, HDL cholesterol, glucose, and insulin. Circulating IL-6 levels did not differ between n-3 PUFA-supplemented non-obese and obese groups (whereas IL-6 levels were significantly increased in obese versus non-obese corn oil groups) Adiponectin levels were decreased in corn oil obese versus non-obese groups, but did not differ between the obese and non-obese fish oil groups
Females with obesity (aged 25–45 years old) [140]	4.8 g of LC n-3 PUFA/day (3.2 g EPA + 1.6 g DHA) for 3 months (*n* = 9)	n-3 PUFA effect compared to baseline (pre-supplementation): ↓ HOMA-IR ↓ plasma insulin ↓ plasma TNF-α Non-change in plasma leptin, IL-6, IL-10, TGF-β, adiponectin, triglycerides and total cholesterol
Females with obesity consuming a very low calorie diet (2200 kJ/day) for 3 weeks (aged ~37–62 years old) [147]	**n-3 PUFA group**: 2.8 g/day of EPA and DHA in a 2:1 ratio for 3 weeks (*n* = 11) **Control group**: saline solution for 3 weeks (*n* = 9)	n-3 PUFA Group: ↑ weight loss ↑ BMI loss ↓ hip circumference No difference in serum fibrinogen (n-3 PUFA versus Control)
Males and females (aged 20–40 years old) with overweight or obesity [148]	**Lean fish consumption** (3 × 150 g portions of cod/week; *n* = 70) **Fatty fish consumption** (3 × 150 g portions of salmon/week; *n* = 74) **Fish oil capsule** (1.5 g/day EPA and DHA) and no seafood consumption (*n* = 68) **Control** (sunflower oil capsule and no seafood consumption; *n* = 66) All groups consumed an energy-restricted diet (30% estimated energy restriction or ~600 kcal/day) for 8 weeks	Modeling approach was used to assess weight loss across intervention diets: Estimated ↑ weight loss in males consuming the fish and fish oil-supplemented diets compared to Control. No significant estimated weight-loss effect of the interventions in females
Males and females with overweight, and ≥1 cardiovascular risk factor: mild hypertension, elevated plasma total cholesterol, or elevated plasma triacylglyceride levels (aged 25–65 years old) [144]	**Fish oil group**: 6 × 1 g tuna fish oil capsules per day containing 250 mg DHA and 60 mg EPA per capsule (1500 mg DHA + 360 mg EPA total n-3 PUFA intake per day) for 12 weeks (*n* = 17) **Placebo group**: 6 g sunflower oil per day for 12 weeks (*n* = 18) Above dietary groups ± exercise (walking 3 day/week for 45 min at 75% age-predicted maximal heart rate) Fish Oil + Exercise (*n* = 16) Placebo + Exercise (*n* = 14)	Fish Oil Group: ↑ plasma HDL cholesterol ↓ plasma triacylglycerides Fish Oil + Exercise Group: ↓ fat mass No significant effect of interventions on body weight
Sedentary males and females with overweight and obesity [149]	**Fish oil group**: 3.0 g EPA plus DHA at a 5:1 ratio (EPA:DHA) per day for 24 weeks (*n* = 64) **Placebo group**: Daily isocaloric soybean and corn oil (1:1 ratio) capsules for 24 weeks (*n* = 64) Both groups exercised aerobically for 150 min/week at 50–85% of their VO_2_ max	No significant difference in the decrease in any anthropometric measurement between groups Both groups lost on average >5% of their body weight, BMI, body fat and waist circumference
Males and females with overweight and obesity (aged 30–75 years old) [150]	**Fish oil group**: 2 g fish oil (containing 640 mg EPA and 480 mg DHA) per day for 6 weeks (*n* = 25) **Control group**: 2 g olive oil capsule per day for 6 weeks (*n* = 25)	Fish Oil Group: ↑ serum adiponectin, ↑ blood glucose No significant difference between groups in BMI, waist circumference, total cholesterol, HDL cholesterol, LDL cholesterol, triglycerides, apolipoprotein-B, serum IL-6, TNF-α or CRP
Males with females with obesity (aged 18–60 years old) [151]	**Fish oil group**: 6 × 1 g tuna fish oil capsule/day containing 70 mg EPA + 270 mg DHA (total daily intake of 420 mg EPA+ 1620 mg DHA) for 14 weeks (*n* = 17) **Placebo group**: 6 × 1 g monounsaturated fatty acid (Sunola oil) capsule/day for 14 weeks (*n* = 12) First 4 weeks of intervention = weight-loss phase (very low energy diet; 3000 kJ/day), followed by a 10-week weight maintenance phase	No significant differences in anthropometric and blood biomarker outcomes between fish oil and placebo groups Fish oil group compared to baseline: ↓ Blood glucose, triacylglycerides, total cholesterol, HDL cholesterol and LDL cholesterol ↓ leptin, TNF-α ↓ Weight, BMI, fat mass, waist and hip circumference
Males with females with obesity (aged 18–60 years old) [152]	Both groups followed an energy-reduced, portion-controlled healthy eating weight-loss diet comprising daily intake of 5000 kJ for females and 6000 kJ for males **Fish oil group**: Tuna oil capsules (6 × 1 g capsule) taken with all meals for a total intake of 1.62 g DHA and 0.42 g EPA per day for 12 weeks (*n* = 15) **Placebo group**: Sunola oil (monounsaturated oil), 6 × 1 g placebo capsule per day for 12 weeks (*n* = 18)	↓ plasma triglycerides in the fish oil group versus placebo No significant differences between the fish oil and placebo groups in anthropometric measurements after 12 weeks of intervention Fish oil group compared to baseline: ↓ Weight, fat mass, BMI, waist and hip circumference ↓ plasma triglycerides ↓ plasma TNF-α
Males and females with obesity that are non-diabetic with either impaired glucose tolerance, impaired fasting glucose, or at least three features of the metabolic syndrome [145]	**Fish oil group** (*n* = 19): 4 g/day omega-3-acid ethyl esters (LOVAZA, fish oil) for 12 weeks **Control group** (*n* = 14): 4 g/day corn oil for 12 weeks	Fish Oil Group compared to baseline: ↓ serum triacylglycerides ↓ plasma MCP-1 No significant differences between fish oil and control groups: weight, insulin sensitivity, circulating total cholesterol, HDL and LDL cholesterol, circulating inflammatory mediators (IL-6, IL-12, TNF-α, resistin, leptin) and circulating adiponectin and IL-10
Females with overweight [146]	Crossover design: 12-week dietary intervention with a 4-week washout between phases **n-3 PUFA** (*n* = 15): 1.3 g EPA and 2.9 g DHA per day **Placebo** (*n* = 18): 2.8 g linoleic acid and 1.4 g oleic acid per day Groups stratified by inflammatory status	In subjects with higher inflammatory status, insulin sensitivity was improved with n-3 PUFA supplementation n-3 PUFA supplementation versus baseline: ↓ triacylglycerides after 4 and 12 weeks ↓ IL-6 and CRP levels after 12 weeks
Adults with obesity and insulin resistance (aged 18–65 years old) [153]	**n-3 PUFA group** (*n* = 12): oral supplement of 4.2 g n-3 PUFA/day (containing 3.9 g EPA + DHA with small amounts of α-linolenic acid, stearidonic acid, etc.) for 6 months **Placebo group** (*n* = 9): 4.2 g/day oleic acid for 6 months	No significant differences between the n-3 PUFA and placebo groups in BMI, fat free mass, % body fat, visceral fat mass
Males and females with overweight or obesity and type 2 diabetes (aged 30–65 years old) [154]	**n-3 PUFA group**: 3 capsules daily containing 300 mg DHA and 600 mg EPA (total n-3 PUFA intake per day: 900 mg DHA and 1800 mg EPA) for 10 weeks **Placebo group**: 3 placebo capsules daily containing paraffin for 10 weeks	n-3 PUFA Group versus Placebo: ↓ HOMA-IR ↑ serum irisin ↓ diastolic blood pressure No significant differences in weight, BMI, waist and hip circumference, insulin, HbA1c
Male and female adolescents (aged 14–17 years old) with obesity [155]	Crossover design with a 3-month intervention followed by a 6-week washout period (males: *n* = 11; females: *n*-14) **n-3 PUFA**: 1.2 g of LC n-3 PUFA per day (930 mg EPA, 290 mg DHA, 100 mg *γ*-linolenic acid) for 3 months **Placebo**: Medium-chain triglyceride capsules for 3 months	n-3 PUFA improved the glucose and insulin response to an intravenous glucose tolerance test in females (but not males) No significant difference between n-3 PUFA and placebo in weight, BMI, hip and waist circumference, fasting circulating lipids (total, LDL and HDL cholesterol, triacylglyceride), glucose, insulin, adiponectin and leptin in males and females

^1^ Arrows indicate direction of change: increase (↑) and decrease (↓).

**Table 2:** Summary of LC n-3 PUFA-mediated effects on obese AT and skeletal muscle function ^1^.

Domain	Effect of LC n-3 PUFA	Key Targets/Markers
Lipid mediators	↑ Pro-resolving mediators	Resolvins, oxylipids
Adipokines	↑ Adiponectin	Adiponectin
	↓ Leptin (variable)	Leptin
	↓ Pro-inflammatory mediators	TNF-α, IL-6, MCP-1
Myokines	↓ Pro-inflammatory mediators	TNF-α, IL-6, MCP-1, MIP-1α, MIP-1β
Inflammatory signaling	↓ NF-κB, STAT3	NF-κB, STAT3
	↓ Inflammasome activity	NLRP3, IL-1β
	↑ Anti-inflammatory signaling	PPARγ, GPR120
	↓ Pro-inflammatory signaling	JNK, MAPK, NF-κB
Immune cell recruitment/chemotaxis	↓ Chemokines	MCP-1, MCP-3, MIP-1α/β, RANTES
Macrophage phenotype	Shift in macrophage polarization: ↓ M1 and ↑ M2	M1 and M2 surface marker expression ↑ IL-10
Glucose metabolism	↑ Glucose uptake	GLUT4 translocation
Systemic metabolism	↓ IR Improved glucose metabolism	Insulin, HOMA-IR

^1^ Arrows indicate direction of change: increase (↑) and decrease (↓).

## Data Availability

No new data were created or analyzed in this study. Data sharing is not applicable to this article.

## Abbreviations

The following abbreviations are used in this manuscript:

Akt	protein kinase B
ALA	α-linolenic acid
AT	adipose tissue
BMI	body mass index
CIDEA	cell death-inducing DFFA-like effector A
CLS	crown-like structures
CRP	C-reactive protein
DAG	Diacylglycerol
DHA	docosahexaenoic acid
EPA	eicosapentaenoic acid
FA	fatty acid
FAP	fibro-adipogenic progenitors
FFA	free fatty acid
GLUT4	glucose transporter type 4
GPR120	G-protein coupled receptor 120
GSK 3	glycogen synthase kinase-3
HbA1c	Hemoglobin A1c
HDL	high-density lipoprotein
HFD	high-fat diet
HOMA-IR	homeostatic model assessment of insulin resistance
IGF	insulin-like growth factor
IL	interleukin
IMCL	intramyocellular lipids
IR	insulin resistance
IRS	insulin receptor substrate
IRS-1	insulin receptor substrate 1
JAK	janus kinases
JNK	c-Jun N-terminal kinase
LC	long chain
LDL	low-density lipoprotein
LPS	lipopolysaccharide
MCP	monocyte chemoattractant protein
MIP	macrophage inflammatory protein
mTORC2	mammalian target of rapamycin complex 2
n-3	omega 3
NFκB	nuclear factor kappa-light-chain-enhancer of activated B cells
NLRP3	NOD-like receptor family pyrin domain containing 3
p38 MAPK	mitogen-activated protein kinase
PGC1α	peroxisome proliferator-activated receptor gamma coactivator 1 alpha
PI3K	Phosphoinositide 3-kinase
PKC-zeta	protein kinase C zeta
PPARγ	peroxisome proliferator-activated receptor gamma
PRDM16	PR domain containing 16
PUFA	polyunsaturated fatty acid
ROS	reactive oxygen species
SFA	saturated fatty acid
SOCS	suppressor of cytokine signaling
STAT	signal transducer and activator of transcription
T2D	type 2 diabetes mellitus
TCA cycle	tricarboxylic acid cycle
TGF-β	transforming growth factor-beta
Th cells	T helper cells
TLR	Toll-like receptor
TNF-α	tumor necrosis factor alpha
UCP1	uncoupling protein 1
VLDL	very-low-density lipoprotein
WAT	white adipose tissue
